# High-resolution crystal structures of *Escherichia coli* FtsZ bound to GDP and GTP

**DOI:** 10.1107/S2053230X20001132

**Published:** 2020-02-05

**Authors:** Maria A. Schumacher, Tomoo Ohashi, Lauren Corbin, Harold P. Erickson

**Affiliations:** a Department of Biochemistry, Duke University School of Medicine, Box 3711, DUMC, Durham, NC 27710, USA; b Department of Cell Biology, Duke University School of Medicine, Box 3711, DUMC, Durham, NC 27710, USA; c Department of Biomedical Engineering, Duke University, Durham, NC 27708, USA

**Keywords:** FtsZ, cytokinesis, treadmilling, protofilaments, Z-ring

## Abstract

Bacterial cytokinesis is mediated by the Z-ring, which is formed by the prokaryotic tubulin homolog FtsZ. Recent data indicate that the Z-ring is composed of small patches of FtsZ protofilaments that travel around the bacterial cell by treadmilling. Treadmilling involves a switch from a relaxed (R) state, favored for monomers, to a tense (T) conformation, which is favored upon association into filaments. The R conformation has been observed in numerous monomeric FtsZ crystal structures and the T conformation in *Staphylococcus aureus* FtsZ crystallized as assembled filaments. However, while *Escherichia coli* has served as a main model system for the study of the Z-ring and the associated divisome, a structure has not yet been reported for *E. coli* FtsZ. To address this gap, structures were determined of the *E. coli* FtsZ mutant FtsZ(L178E) with GDP and GTP bound to 1.35 and 1.40 Å resolution, respectively. The *E. coli* FtsZ(L178E) structures both crystallized as straight filaments with subunits in the R conformation. These high-resolution structures can be employed to facilitate experimental cell-division studies and their interpretation in *E. coli*.

## Abstract 2

*Escherichia coli* has served as the main model system for examining cell division in bacteria; however, the structure of the central cytokinesis protein FtsZ has not been determined to date. Here, high-resolution structures of *E. coli* FtsZ in GDP-bound and GTP-bound states are described.

## 1. Introduction   

Cytokinesis, the process by which a cell divides into daughter cells, is essential for all life. Bacteria accomplish this task by employing a multiprotein complex called the divisome. Work over the past three decades has witnessed the discovery and characterization of most of the divisome proteins in *Escherichia coli* (Margolin, 2005 ▸; Adams & Errington, 2009 ▸; Rowlett & Margolin, 2015 ▸; Tsang & Bernhardt, 2015 ▸; Du & Lutkenhaus, 2017 ▸; den Blaauwen *et al.*, 2017 ▸; Lutkenhaus *et al.*, 2017 ▸; Söderström & Daley, 2017 ▸). FtsZ, a bacterial homolog of tubulin, assembles onto a ring-like scaffold called the Z-ring, which serves as the foundation for cell division by the divisome (Bi & Lutkenhaus, 1991 ▸; Nogales *et al.*, 1998 ▸; Erickson *et al.*, 2010 ▸; Haeusser & Margolin, 2016 ▸; Du & Lutkenhaus, 2019 ▸). FtsZ assembles into single-stranded protofilaments, and these associate into clusters that are anchored to the cell membrane via a disordered linker and a C-terminal peptide that attaches to FtsA, ZipA and/or other proteins (Ma *et al.*, 1996 ▸; Pichoff & Lutkenhaus, 2005 ▸; Gardner *et al.*, 2013 ▸; Buske & Levin, 2013 ▸; Sundararajan *et al.*, 2015 ▸). Recent work has shown that clusters of membrane-bound FtsZ move around the circumference of the cell by treadmilling, and that these moving patches carry with them the divisome cell-wall re­modeling machinery (Strauss *et al.*, 2012 ▸; Rowlett & Margolin, 2014 ▸; Bisson-Filho *et al.*, 2017 ▸; Yang *et al.*, 2017 ▸; Perez *et al.*, 2019 ▸; Li *et al.*, 2018 ▸; Söderström *et al.*, 2019 ▸). FtsZ treadmilling has also been visualized *in vitro* on supported lipid bilayers (Loose & Mitchison, 2014 ▸; Ramirez-Diaz *et al.*, 2018 ▸). Treadmilling is now established as the primary mechanism for FtsZ assembly dynamics.

Apart from treadmilling at steady state, FtsZ assembly *in vitro* exhibits two aspects of cooperative assembly: an un­favorable nucleation step and a critical concentration. However, this raises the question of how a single-stranded protofilament with only one type of bond can exhibit cooperative assembly. Several groups have suggested that this could be achieved if FtsZ had two conformations, one with a high and one with a low affinity for making longitudinal bonds (Michie & Löwe, 2006 ▸; Dajkovic & Lutkenhaus, 2006 ▸; Dajkovic *et al.*, 2008 ▸; Huecas *et al.*, 2008 ▸; Miraldi *et al.*, 2008 ▸). To explain the cooperativity, the model proposes that FtsZ monomers are highly favored to be in the low-affinity conformation, whereas assembly into a protofilament switches the subunit to favor the high-affinity conformation (Michie & Löwe, 2006 ▸; Dajkovic & Lutkenhaus, 2006 ▸; Dajkovic *et al.*, 2008 ▸; Huecas *et al.*, 2008 ▸; Miraldi *et al.*, 2008 ▸).

Prior to 2012, numerous FtsZ crystal structures were solved from different bacterial species in GTP-bound, GDP-bound or apo states (Löwe & Amos, 1998 ▸; Leung *et al.*, 2000 ▸; Cordell *et al.*, 2003 ▸; Oliva *et al.*, 2004 ▸, 2007 ▸). These structures all had the same basic structure: two globular subdomains connected by a long helix, H7. The N-terminal subdomain contains the binding site for GTP, and the C-terminal subdomain has, by analogy with tubulin protofilaments, key catalytic residues that contact the GTP in the subunit below to initiate GTP hydrolysis (Nogales *et al.*, 1998 ▸; Erickson, 1998 ▸; Scheffers *et al.*, 2002 ▸). Importantly, these structures were mostly of FtsZ monomers.

A major advance in our understanding of FtsZ structure and polymerization was achieved when two laboratories independently crystallized FtsZ from *Staphylococcus aureus* (SaFtsZ) and found that it crystallized in the form of long, straight protofilaments (Matsui *et al.*, 2012 ▸; Elsen *et al.*, 2012 ▸). The FtsZ in these protofilaments showed a striking conformational change relative to the previous monomeric forms, even the closely related *Bacillus subtilis* FtsZ (BsFtsZ) (Oliva *et al.*, 2007 ▸; Matsui *et al.*, 2012 ▸; Elsen *et al.*, 2012 ▸). As noted, the globular domain of both FtsZ and tubulin is divided into two subdomains. The conformational change from BsFtsZ to SaFtsZ involved a large rotation (25–28°) of the C-terminal subdomain relative to the N-terminal subdomain. Accompanying this rotation, the helix H7 shifted downwards by a turn (Matsui *et al.*, 2012 ▸; Elsen *et al.*, 2012 ▸). This new structure of SaFtsZ assembled in straight protofilaments with a large interface and was an excellent candidate for the high-affinity, protofilament-forming conformation, while the monomeric BsFtsZ represented the low-affinity conformation. The two conformations of FtsZ have been designated T (tense) for the high-affinity state and R (relaxed) for the low-affinity state (Matsui *et al.*, 2014 ▸; Fujita *et al.*, 2017 ▸). Alternatively, the two conformations have been designated open and closed, referring to the cleft between the two subdomains (Wagstaff *et al.*, 2017 ▸). We will adopt the R and T nomenclature here.

Although wild-type SaFtsZ assembles preferentially into protofilaments with subunits in the T conformation, several mutants of SaFtsZ have been found assembled into protofilaments with subunits in the R conformation (Matsui *et al.*, 2014 ▸; Wagstaff *et al.*, 2017 ▸). Interestingly, Fujita and coworkers obtained a crystal of wild-type SaFtsZ containing subunits in both R and T states (Fujita *et al.*, 2017 ▸). Apparently, there is only a small free-energy difference between the R and T conformations when SaFtsZ subunits are assembled into protofilaments under crystallization conditions.

Strikingly, although *E. coli* has provided the most detailed understanding of the machinery and mechanism of bacterial cell division, the structure of the *E. coli* FtsZ (EcFtsZ) protein has remained elusive. A likely problem is the tendency of EcFtsZ to aggregate under non-assembly conditions (Mukherjee *et al.*, 1993 ▸). Du and coworkers provided a potentially important advance when they explored several EcFtsZ mutants that had previously been identified as defective in assembly (Du *et al.*, 2015 ▸; Li *et al.*, 2013 ▸). Du and coworkers focused on one mutant, L178E, which they confirmed to be assembly-incompetent in the presence of GTP. They also found that EcFtsZ(L178E) lost its tendency to aggregate when bound to GDP, as determined by size-exclusion chromatography (Du *et al.*, 2015 ▸). This suggested that this mutant would be a good candidate for crystallo­graphy, and indeed they reported that ‘FtsZ-L178E is self-interacting at higher concentrations since it assembled into protofilaments when crystallized’ (Du *et al.*, 2015 ▸). However, these crystals were apparently not suitable for effective structure determination; the structure was never described nor was it deposited in the Protein Data Bank (Joe Lutkenhaus, personal communication). Thus, we decided to address this gap and repeated crystallographic screens in order to obtain better crystals. Here, we report the 1.35 and 1.40 Å resolution structures of *E. coli* FtsZ(L178E) bound to GDP and GTP, respectively.

## 2. Experimental   

### 2.1. Production and purification of *E. coli* FtsZ(L178E)   

The pET-11 vector encoding the *E. coli* K-12 *ftsZ* gene was originally obtained from Bramhill and Thompson (Bramhill & Thompson, 1994 ▸). Truncation and site-directed mutants were generated from the vector and were also expressed in pET-11. The *E. coli* FtsZ truncation mutant used in this study encodes residues 10–316 of the protein. Mutants, *ftsZ(L178E), ftsZ(L178E/M206E)* and *ftsZ(L178E/L272E)*, were also generated using QuikChange (Agilent) in the context of the truncated protein. *E. coli* BL21(DE3) cells were transformed with these expression vectors. Cells expressing each mutant protein were grown to an OD_600_ of ∼1.2 and induced by adding 0.5 m*M* isopropyl β-d-1-thiogalatopyranoside for 3 h at 37°C. After induction, the cells were centrifuged for 45 min at 15 000*g*. The resultant cell pellets were frozen at −80°C until use. Frozen cells were suspended in resuspension buffer consisting of 50 m*M* Tris–HCl pH 7.9, 100 m*M* NaCl, 2 m*M* phenylmethylsulfonyl fluoride, and 0.75 mg ml^−1^ lysozyme was added. After 30 min on ice the cells were refrozen, thawed and lysed by sonication. The cells were sonicated eight times for 30 s on ice. The lysate was centrifuged at 80 000*g* for 30 min and the supernatant was recovered. The soluble bacterial proteins were first precipitated with 50% saturated ammonium sulfate. The resuspended proteins were then dialyzed against a buffer composed of 50 m*M* Tris–HCl pH 7.9, 50 m*M* KCl, 10%(*v*/*v*) glycerol, 1 m*M* EDTA. The proteins were purified by chromatography on a Resource Q anion-exchange column (GE Healthcare) with a linear gradient from 50 to 500 m*M* KCl. Fractions containing protein were identified by SDS–PAGE analysis, and those containing protein were pooled and the protein was concentrated using a Microcon 30 kDa cutoff concentrator (Millipore). The FtsZ protein concentrations were determined as described previously using bicinchoninic acid colorimetric assays (Lu *et al.*, 1998 ▸).

### 2.2. Crystallization of *E. coli* FtsZ(L178E)–GDP and *E. coli* FtsZ(L178E)–GTP complexes   

Full-length, truncated and truncated mutant FtsZ proteins were used in crystallization trials. The EcFtsZ(L178E) protein truncated to the globular domain from residues 10 to 316 produced well diffracting crystals and thus was used for structural studies [and will be referred to here as EcFtsZ(L178E)]. To obtain crystals of EcFtsZ(L178E) with GDP, the protein was concentrated to 37 mg ml^−1^ and GDP was added to the solution, resulting in a final concentration of 1 m*M*. The mixture was centrifuged for 3 s at 20 000*g* and then used in hanging-drop vapor-diffusion crystallization screens. The screens employed were Wizard Classic 1–4 (Rigaku), Wizard Cryo 1 and Cryo 2 (Rigaku), PEGRx 1 and PEGRx 2 (Hampton Research), Crystal Screen (Hampton Research), SaltRx 1 and SaltRx 2 (Hampton Research). The screens were all performed at room temperature using 24-well plates. In the screens, 1 µl protein solution was mixed with 1 µl cystallization reagent and placed over the reservoir. The most promising crystals were obtained with Wizard Classic 3 condition 35. This condition was further optimized and the best crystals were obtained by combining 5 µl EcFtsZ(L178E)–GDP complex with 5 µl crystallization solution consisting of 50 m*M* sodium cacodylate pH 6.5, 11%(*w*/*v*) PEG 8000, 150 m*M* calcium acetate, 20%(*v*/*v*) glycerol and hanging the drop over a reservoir containing 750 µl crystallization solution (Table 1 ▸). The crystals grew overnight and reached a maximum size of 0.3 × 0.5 × 0.8 mm over 1–1.5 weeks. The EcFtsZ(L178E)–GTP crystals were obtained similarly using protein at 30 mg ml^−1^ with a final concentration of 1 m*M* GTP (Table 1 ▸). These crystals were isomorphous to the EcFtsZ(L678E)–GDP crystals. The EcFtsZ(L178E)–GTP complex was screened (at room temperature) using all of the commercial screens used for the EcFtsZ(L178E)–GDP complex. The best crystals of the EcFtsZ(L178E)–GTP complex were also obtained with Wizard Classic 3 condition 35. Optimal crystals were obtained by mixing the complex in a 1:1 ratio with a reservoir consisting of 13%(*w*/*v*) PEG 8000, 50 m*M* sodium cacodylate pH 6.5, 150 m*M* calcium acetate, 20%(*v*/*v*) glycerol and hanging the drop over a reservoir containing 750 µl crystallization reagent (Table 1 ▸). The crystals grew overnight and reached similar sizes to the EcFtsZ(L178E)–GDP crystals in 1–2 weeks. Both crystals were cryopreserved directly from the crystallization solution, which acted as a cryosolvent. Data were collected on beamline 8.3.1 at the Advanced Light Source (ALS), USA. The crystals were looped and placed directly in the liquid-nitrogen stream in the hutch and data were collected.

### 2.3. Crystal structure determination of the *E. coli* FtsZ(L178E)–GDP and *E. coli* FtsZ(L178E)–GTP complexes   

X-ray intensity diffraction data for the EcFtsZ(L178E)–GDP and EcFtsZ(L178E)–GTP complexes were collected on beamline 8.3.1 at the ALS. The data were processed using *MOSFLM* and scaled with *SCALA* (Leslie, 2006 ▸; Potterton *et al.*, 2003 ▸; Table 2 ▸). Both the EcFtsZ(L178E)–GDP and EcFtsZ(L178E)–GTP crystals belonged to the monoclinic space group *P*2_1_. Molecular replacement (MR) using the *Pseudomonas aeruginosa* FtsZ structure (PDB entry 2vaw; Oliva *et al.*, 2007 ▸) without the GDP and waters as a model was successful in solving the EcFtsZ(L178E)–GDP structure using *MOLREP* and *Phaser* (Vagin & Teplyakov, 2010 ▸; McCoy *et al.*, 2007 ▸). Both produced clear solutions [with a log likelihood gain (LLG) of 309.67 in *Phaser*]. There is one EcFtsZ(L178E) protein molecule in the crystallographic asymmetric unit. After an initial round of refinement in *Phenix* (Liebschner *et al.*, 2019 ▸) the residues that were not identical between *P. aeruginosa* and *E. coli* were replaced by their *E. coli* counterparts. This structure was then subjected to multiple iterative cycles of rebuilding in *O* and refinement in *Phenix* (Jones *et al.*, 1991 ▸; Liebschner *et al.*, 2019 ▸). GDP and water molecules were added in the final stages of refinement. The final model included EcFtsZ(L178E) residues 10–65, 67–169 and 173–316, one GDP molecule and 339 water molecules. Residues 63–73, 166–176 and 301–303 harbored weak electron density. The EcFtsZ(L178E)–GTP data were isomorphous with the EcFtsZL178E)–GDP data. However, in order to ensure an optimal starting model, MR was used with the EcFtsZ(L178E)–GDP structure minus the GDP as a search model in *Phaser*, which resulted in a solution with an LLG of 15 026. The final EcFtsZ(L178E)–GTP model included EcFtsZ(L178E) residues 10–169 and 173–316, one GTP molecule and 339 water molecules. Similar to the EcFtsZ(L178E)–GDP structure, weak electron density was observed for residues 62–73, 165–176 and 301–303 in this structure. See Tables 2 ▸ and 3 ▸ for data-collection and refinement statistics.

## 3. Results and discussion   

### 3.1. *E. coli* FtsZ(L178E)–GDP and *E. coli* FtsZ(L178E)–GTP structure determination   

**Figure 1:**
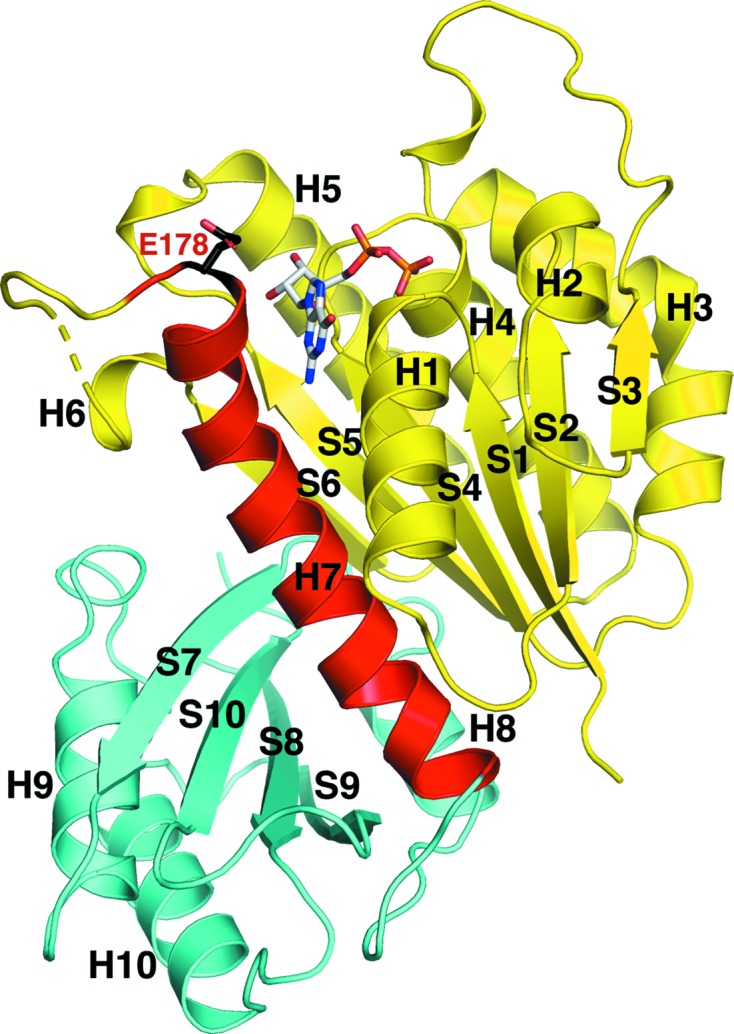
Overall structure of the EcFtsZ(L178E)–GDP complex. The protein is shown as a ribbon diagram with the N-terminal subdomain (residues 9–178) colored yellow, the connecting H7 helix colored red and the C-­terminal subdomain (residues 201–216) colored cyan. S and H indicate strands and helices, respectively. The bound GDP is shown as sticks. Also shown as sticks is residue Glu178, the site of the mutation. The structure is labeled according to the common nomenclature for the tubulin/FtsZ family of GTPases (Nogales *et al.*, 1998 ▸). All ribbon diagrams were made using *PyMOL* (DeLano, 2002 ▸).

The EcFtsZ(L178E) protein, which previous studies had indicated to be a monomer in the presence of GDP or GTP (Du *et al.*, 2015 ▸), produced well diffracting crystals in the presence of GDP and, when optimized, permitted us to collect data to 1.35 Å resolution. As the *P. aeruginosa* FtsZ protein is the most similar in amino-acid sequence to EcFtsZ (overall identity of 67% over the conserved core of our truncated protein), we used the *P. aeruginosa* FtsZ–GDP structure (PDB entry 2vaw; Oliva *et al.*, 2007 ▸) as a search model for molecular replacement. One clear solution was obtained, consistent with the presence of one EcFtsZ(L178E) molecule in the crystallographic asymmetric unit. Clear electron density was observed in the structure for GDP (Figs. 1 ▸ and 2 ▸ *a*). After the addition of the GDP and water molecules and further refinement, the structure converged to final *R*_cryst_ and *R*_free_ values of 18.6% and 20.4%, respectively, to 1.35 Å resolution.

**Figure 2:**
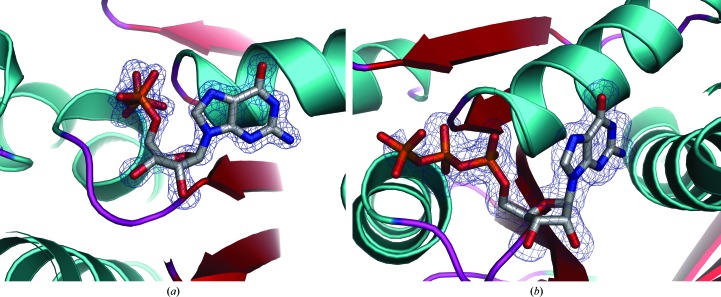
OMIT maps of GDP and GTP in EcFtsZ(L178E) structures. (*a*) Close-up of the GDP bound in the N-terminal subdomain pocket in EcFtsZ(L178E). The helices, strands and loops are colored cyan, red and magenta, respectively. The blue mesh corresponds to the *F*_o_ − *F*_c_ OMIT map (in which the GDP was removed) contoured at 3.5σ around the GDP. (*b*) Close-up of the GTP in the EcFtsZ(L178E)–GTP complex with the protein secondary-structural elements colored as in Fig. 2 ▸(*a*). The *F*_o_ − *F*_c_ map is shown as a blue mesh and is contoured at 2.9σ.

The GTPase activity of FtsZ is largely dependent on polymerization, as the GTP-binding pocket at the ‘top’ of a subunit must make contact with the catalytic T7 loop residues at the bottom of an adjacent subunit in the protofilament to hydrolyze the bound GTP (Erickson, 1998 ▸; Scheffers *et al.*, 2002 ▸). Because EcFtsZ(L178E) has been shown to be deficient in GTPase activity owing to its inability to form filaments (Du *et al.*, 2015 ▸), we considered that it was likely that it would crystallize with GTP. Crystals were successfully obtained of the EcFtsZ(L178E)–GTP complex that were isomorphous with the EcFtsZ(L178E)–GDP crystals. The EcFtsZ(L178)–GTP structure was solved and refined to final *R*_cryst_ and *R*_free_ values of 18.5% and 20.5%, respectively, to 1.40 Å resolution. Notably, the electron density for the γ-phosphate moiety of the GTP in the EcFtsZ(L178E)–GTP structure was weak (Fig. 2 ▸ *b*); the average *B* factor for the atoms of the γ-phosphate is 63.1 Å^2^ compared with 19.2 Å^2^ for the remaining atoms of the GTP. These data suggest that this mutant may retain residual GTPase activity or that the γ-phosphate is disordered in the structure.

### 3.2. *E. coli* FtsZ(L178E) crystallizes in the form of straight protofilaments   

**Figure 3:**
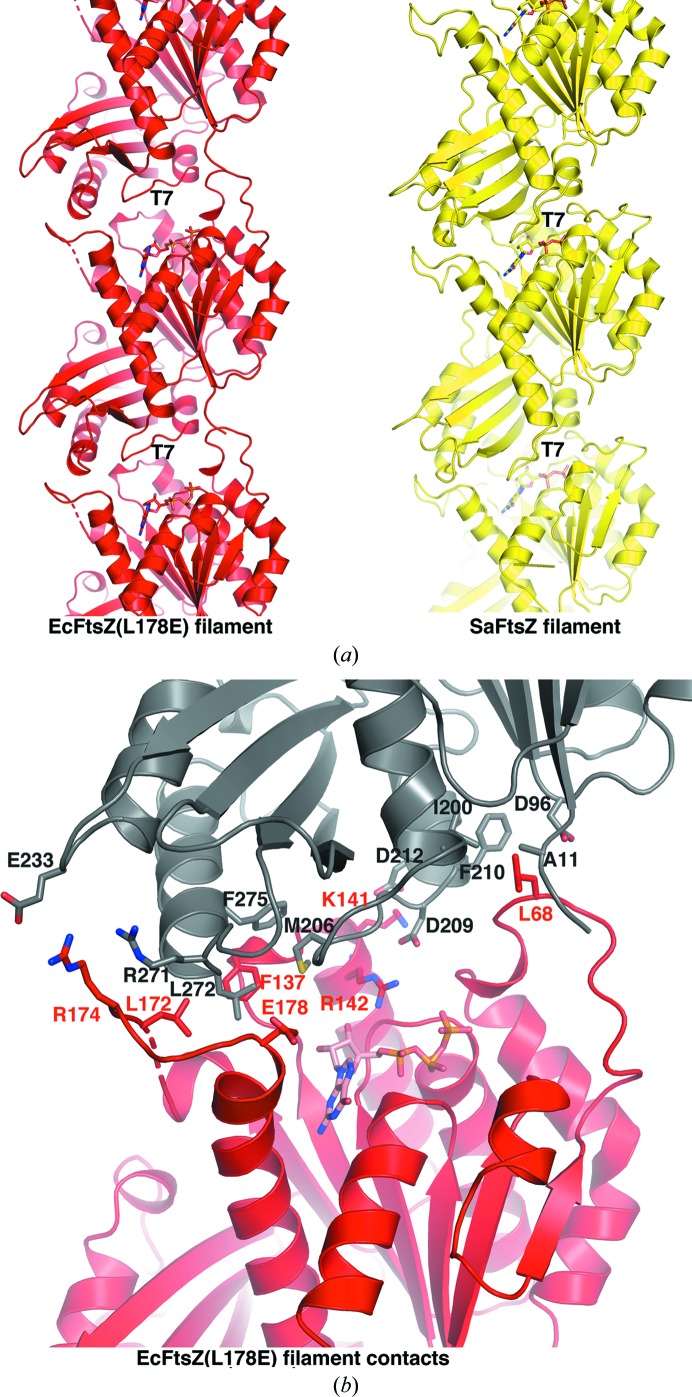
The EcFtsZ(L178E) filament. (*a*) Side-by-side comparison (after aligning the middle subunit) of the EcFtsZ(L178E) filament (red) generated by its crystal structure packing and the filament observed in the T-state SaFtsZ structure (PDB entry 3voa; yellow). The position of the T7 loop in each subunit is labeled, showing that this key catalytic loop is not properly positioned in the EcFtsZ filament (next to the GTP) as in the T-state filament. (*b*) Close-up view of the intersubunit contacts in the EcFtsZ(L178E) filament.

In spite of evidence that the L178E mutation in EcFtsZ inhibits assembly, the crystals showed that EcFtsZ(L178E) assembled into straight protofilaments, as noted in the previous study of Du *et al.* (2015 ▸). However, comparison of the EcFtsZ(L178E) filament with the SaFtsZ filament reveals that the EcFtsZ filament is much looser (see below for the buried surface areas). While the residues in the EcFtsZ(L178E) loops from residues 63 to 70 and 168 to 178 make somewhat similar interactions with neighboring subunits to those observed in the SaFtsZ filament, they are more surface-exposed in the EcFtsZ(L178E) crystal filament and their relatively high *B* factors (the average *B* factors for residues 63–70 and residues 168–178 are 56.9 and 45.5 Å^2^, respectively, compared with the overall average protein *B* factor of 23.9 Å^2^) indicate they are somewhat flexible. In addition, the electron density for these residues is sparse. Presumably, as the residue corresponding to Leu178 in SaFtsZ is wedged into a hydrophobic pocket of an adjacent subunit in the SaFtsZ filament, the similar burial of the Glu178 side chain into the hydrophobic pocket in the EcFtsZ(L178E) filament is predicted to be unfavorable. As a result, the EcFtsZ(L178E) protofilaments have a more open subunit interface. In this filament, Leu68 from the loop connecting S3 and H3 is inserted into a hydrophobic pocket composed of Ala11, the C^β^ atom of Asp96, Ile200 and Phe210 of the adjacent subunit (Fig. 3 ▸ *b*). Leu68 has an elevated average *B* factor (48.6 Å^2^) compared with the overall *B* factor of 23.9 Å^2^ for the protein, suggesting that the contact might be weak. In the EcFtsZ(L178E) loop connecting H6 and H7, which contains the L178E mutation, Arg174 makes electrostatic interactions with Glu233 and stacks with the Arg271 side chain from the adjacent subunit, and Leu172 from the loop makes weak van der Waals interactions with the side chain of Leu272 from the neighboring subunit (Fig. 3 ▸ *b*). Next to this loop, Phe137 also makes contacts with Leu272 and also Met206 and Phe275 from the adjacent subunit (Fig. 3 ▸ *b*). Other cross-contacts include electrostatic interactions between Lys141 and Arg142 and the neighboring subunit residues Asp209 and Asp212, respectively. Consistent with the finding that the EcFtsZ(L178E) filament has fewer contacts between subunits, the buried surface area (BSA) between subunits in the interface of the EcFtsZ(L178E) protofilament is only 720 Å^2^, compared with the BSA of ∼1200 Å^2^ observed in the snug interface between subunits in the SaFtsZ filament (Krissinel & Henrick, 2007 ▸; Matsui *et al.*, 2012 ▸; Fig. 3 ▸ *a*). This is similar to the BSA of 740–810 Å^2^ that Matsui and coworkers observed for SaFtsZ mutants that polymerized in the R form (Matsui *et al.*, 2014 ▸).

Further supporting the notion that the EcFtsZ(L178E) filament is not the form found in the Z-ring, the longitudinal spacing of subunits in the crystallized protofilament is 47.0 Å, which is significantly larger than the value of 42–43 Å measured for EcFtsZ protofilaments by negative-stain EM (Erickson *et al.*, 1996 ▸), the value of 44.0 Å observed in the SaFtZ protofilaments in crystals (Matsui *et al.*, 2012 ▸) and the value of ∼40 Å measured by cryo-EM under native-like conditions (Szwedziak *et al.*, 2014 ▸). Also in this filament, the T7 loops are not properly positioned to interact with the GTP of the subunit below (Fig. 3 ▸ *a*). However, we note that this filament could represent an intermediate in the assembly or disassembly of functional filaments.

### 3.3. *E. coli* FtsZ(L178E) structures are in the R conformation   

**Figure 4:**
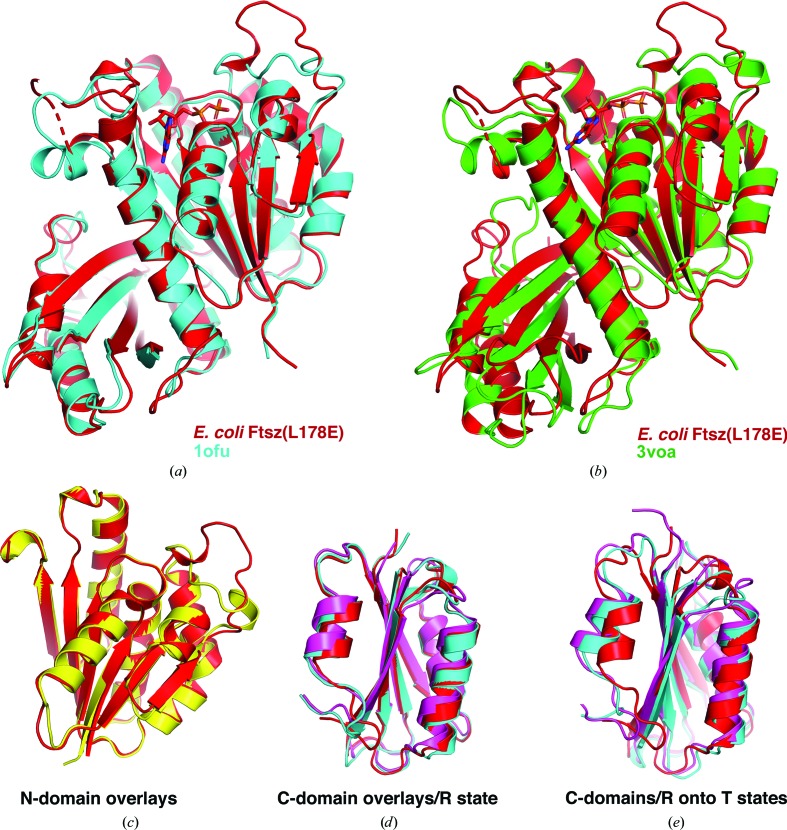
The EcFtsZ(L178E) structure adopts the R state. (*a*) Superimposition of the EcFtsZ(L178E) structure (red) onto the R-state *S. aureus* FtsZ structure (PDB entry 1ofu; cyan). (*b*) Overlay of the EcFtsZ(L178E) structure (red) onto the T-state *S. aureus* FtsZ structure (PDB entry 3voa; green). (*c*) Overlay of the EcFtsZ(L178E) N-terminal subdomain (red) onto the N-terminal subdomain of the T-state structure (PDB entry 3voa; yellow), showing that the N-terminal subdomains adopt the same conformations in R and T states. (*d*) Superimposition of the C-terminal subdomain of EcFtsZ(L178E) (red) onto the C-terminal subdomain of the R-state FtsZ structures (PDB entry 3wgl, magenta; PDB entry 1ofu, cyan), showing they have the same general fold (r.m.s.d.s of 1.1 and 0.8 Å, respectively). (*e*) Superimposition of the EcFtsZ(L178E) C-terminal subdomain (red) onto the C-terminal subdomains of the T-state FtsZ structures (PDB entry 4dxd, cyan; PDB entry 3voa, magenta), showing that the EcFtsZ (R-state) C-terminal subdomain has structural differences compared with T-state C-terminal subdomains (both r.m.s.d.s are >2 Å).

*DALI* structural homology searches revealed that the FtsZ structures with the strongest similarity to EcFtsZ(L178E) are the *P. aeruginosa* FtsZ (PDB entry 1ofu; Cordell *et al.*, 2003 ▸) and *S. aureus* FtsZ (PDB entry 3wgl; Matsui *et al.*, 2014 ▸) structures. PDB entry 1ofu is the structure of *P. aeruginosa* FtsZ bound to SulA (Cordell *et al.*, 2003 ▸), and 293 of the C^α^ atoms in the FtsZ subunit of this structure could be superimposed onto the corresponding atoms in the EcFtsZ(L178E) structure with an r.m.s.d. of 1.1 Å (Fig. 4 ▸ *a*). The structure with PDB code 3wgl contains an *S. aureus* mutant in which the SGEVN sequence of the T7 loop was replaced by a shorter GAN sequence (Matsui *et al.*, 2014 ▸). This FtsZ mutant was crystallized with GDP to produce the structure deposited as PDB entry 3wgl. Despite the fact that *S. aureus* FtsZ shows 14% less sequence identity to the *E. coli* protein compared with *P. aeruginosa* FtsZ, PDB entry 3wgl shows essentially the same degree of structural homology; 289 C^α^ atoms of the 3wgl *S. aureus* FtsZ structure could be superimposed onto the corresponding C^α^ atoms of the EcFtsZ(L178E) structure with an r.m.s.d. of 1.1 Å. Importantly, both PDB entries 1ofu and 3wgl are in the R conformation, supporting the adoption of the R state by EcFtsZ(L178E) in the crystal. Indeed, superimposition of the EcFtsZ(L178E) structure onto the T-state conformation of FtsZ (PDB entry 3voa; Matsui *et al.*, 2012 ▸) resulted in a large r.m.s.d. of 2.3 Å for 269 corresponding C^α^ atoms (Fig. 4 ▸ *b*).

### 3.4. C-terminal subdomain structural differences revealed in filament compared with nonfilament FtsZ   

Our analyses show that the EcFtsZ(L178E) structure adopts the R state and as such has a closed cleft between its N-terminal and C-terminal subdomains (Wagstaff *et al.*, 2017 ▸). When we compared the structure of the N-terminal sub­domain of the EcFtsZ(L178E) structure with the N-terminal subdomain of both R-state and T-state FtsZ structures, we found they are all very similar; they all overlay with r.m.s.d.s of <1 Å (Fig. 4 ▸ *c*). Interestingly, although the C-terminal sub­domain of EcFtsZ(L178E) is structurally similar to those of other R-state structures (r.m.s.d.s of between 0.8 and 1.2 Å; Fig. 4 ▸ *d*), T-state C-terminal subdomain structures showed significant structural differences from the EcFtsZ(L178E) C-terminal subdomain and thus other R-state C-terminal subdomains, with r.m.s.d.s of >2.1 Å for these overlays (Fig. 4 ▸ *e*). A main difference in these C-terminal subdomain structures appears to be in the region encompassing the T7 loop and helix H8. The relevance of this structural difference between the C-terminal subdomains of the T and R states of FtsZ, if any, remains to be determined.

## 4. Conclusions   

Studies in the last several decades using *E. coli* have revealed the machinery of the cell-division divisome and its association with the FtsZ Z-ring. A plethora of regulatory proteins that interact with *E. coli* FtsZ to affect its ability to form a Z-ring and its localization in the cell have also been discovered (Ortiz *et al.*, 2016 ▸). However, to date a structure of EcFtsZ has not been reported. Here, we describe high-resolution structures of the EcFtsZ(L178E) protein in complex with GDP and GTP. These structures also allow the interpretation of multiple mutations that have been constructed and tested for their effects on cell division in this protein (Lu *et al.*, 2001 ▸; Stricker & Erickson, 2003 ▸; Addinall *et al.*, 2005 ▸; Redick *et al.*, 2005 ▸; Osawa *et al.*, 2008 ▸; Haeusser *et al.*, 2015 ▸; Márquez *et al.*, 2017 ▸) and can serve as the foundation for the design of future cell-division experiments in *E. coli* to assess the role of specific residues in the function of FtsZ as well as its interactions with regulatory proteins.

## Supplementary Material

PDB reference: *E. coli* FtsZ(L178E), complex with GDP, 6umk

PDB reference: complex with GTP, 6unx

## Figures and Tables

**Table 1:** Crystallization conditions for EcFtsZ(L178E)–nucleotide structures

	EcFtZ(L178E)–GDP	EcFtsZ(L178E)–GTP
Method	Hanging-drop vapor diffusion	Hanging-drop vapor diffusion
Plate type	24-well plates	24-well plates
Temperature (K)	298	298
Protein concentration (mg ml^−1^)	37	30
Buffer composition of protein solution	50 m*M* Tris–HCl pH 7.9, 50 m*M* KCl, 10% glycerol, 1 m*M* EDTA	50 m*M* Tris–HCl pH 7.9, 50 m*M* KCl, 10% glycerol, 1 m*M* EDTA
Composition of reservoir solution	50 m*M* sodium cacodylate pH 6.5, 11% PEG 8000, 150 m*M* calcium acetate	50 m*M* sodium cacodylate pH 6.5, 13% PEG 8000, 150 m*M* calcium acetate
Volume and ratio of drop	5 µl:5 µl	5 µl:5 µl
Volume of reservoir (ml)	0.75	0.75

**Table 2:** Data collection and processing Values in parentheses are for the highest resolution shell.

	EcFtsZ(L178E)–GDP	EcFtsZ(L178E)–GTP
Source	Beamline 8.3.1, ALS	Beamline 8.3.1, ALS
Wavelength (Å)	1.01	1.01
Temperature (K)	100	100
Detector	PILATUS3 S 6M	PILATUS3 S 6M
Space group	*P*2_1_	*P*2_1_
*a*, *b*, *c* (Å)	37.4, 85.2, 41.4	37.3, 85.1, 41.3
α, β, γ (°)	90.0, 106.2, 90.0	90.0, 107.0, 90.0
Mosaicity (°)	0.20	0.55
Resolution range (Å)	35.90–1.35	42.57–1.40
Total No. of reflections	167120	134137
No. of unique reflections	52792	41849
Completeness (%)	96.7 (86.6)	86.3 (81.0)
Multiplicity	3.1 (2.4)	3.0 (2.0)
〈*I*/σ(*I*)〉	23.8 (5.8)	15.1 (4.1)
*R*_merge_	0.031 (0.069)	0.042 (0.125)
*R*_p.i.m._	0.021 (0.047)	0.025 (0.105)
CC_1/2_	0.998 (0.995)	0.998 (0.990)
Overall *B* factor from Wilson plot (Å^2^)	13.6	13.9

**Table 3:** Structure refinement Values in parentheses are for the highest resolution shell.

Structure	EcFtsZ(L178E)–GDP (PDB code 6umk)	EcFtsZ(L178E)–GTP (PDB code 6unx)
Resolution range (Å)	35.90–1.35	42.57–1.40
Final *R*_cryst_	0.186	0.185
Final *R*_free_	0.204	0.205
No. of non-H atoms		
Protein	2176	2198
Ligand	28	32
Water	303	304
R.m.s. deviations		
Bonds (Å)	0.98	1.02
Angles (°)	0.007	0.007
Average *B* factors (Å^2^)		
Protein	23.9	25.4
Ligand	18.3	24.1
Solvent	33.3	34.5
Ramachandran plot		
Favored regions (%)	99.0	98.3
Allowed (%)	1.0	1.7
Disallowed (%)	0.0	0.0
